# Lignin and Lignin-Derived Compounds for Wood Applications—A Review

**DOI:** 10.3390/molecules26092533

**Published:** 2021-04-26

**Authors:** Johannes Karthäuser, Vladimirs Biziks, Carsten Mai, Holger Militz

**Affiliations:** Department of Wood Biology and Wood Products, Georg-August University of Goettingen, Büsgenweg 4, 37077 Göttingen, Germany; vbiziks@gwdg.de (V.B.); cmai@gwdg.de (C.M.); hmilitz@gwdg.de (H.M.)

**Keywords:** lignin, adhesive, LPF resins, wood modification

## Abstract

Improving the environmental performance of resins in wood treatment by using renewable chemicals has been a topic of interest for a long time. At the same time, lignin, the second most abundant biomass on earth, is produced in large scale as a side product and mainly used energetically. The use of lignin in wood adhesives or for wood modification has received a lot of scientific attention. Despite this, there are only few lignin-derived wood products commercially available. This review provides a summary of the research on lignin application in wood adhesives, as well as for wood modification. The research on the use of uncleaved lignin and of cleavage products of lignin is reviewed. Finally, the current state of the art of commercialization of lignin-derived wood products is presented.

## 1. Introduction

Lignin is the most abundant aromatic polymer in nature and the second most abundant biomass on earth [1,2,3]. In paper production and other processes, lignin is obtained as a side product [4]. Over 50 million tons of lignin are currently annually produced as a side product in the paper industry alone. Additionally, the amount of isolated lignin produced is expected to increase [5]. Due to the recalcitrant, heterogeneous and complicated structure it is difficult to apply the thus-produced lignin, which is the reason why about 95% of it is burned to regain the inorganic chemicals necessary for the pulping process and to supply energy [6,7,8].

Because of the described situation and the phenolic-rich structure of the lignin, the conversion of technical lignin to higher-value products has been a topic of interest for many years. Despite major research and efforts, industrial applications of lignin are still only the exception [9,10]. Examples of the industrial usage of lignin are the production of vanillin or the addition of lignin to concrete, as well as dispersing and binding agents [9,11]. Additionally, several companies are selling purified lignin [12,13,14].

Due to the high phenolic content and the ability to form solid networks, the application of lignin for the treatment of wood is of interest. The phenolic structure of lignin leads to the idea of substituting phenol, which is conventionally won from non-renewable resources, in phenol-formaldehyde (PF) resins [15]. A lot of research has been conducted to implement lignin into the resins and to modify the lignin to improve its feasibility [16,17]. However, until now there are only a few companies that offer wood products based on lignin [15,18]. Hence, the research interest is still high, and potential applications are by now not only in the substitution for phenol in PF resins, but also in the substitution for urea in urea-formaldehyde (UF) and a broad range of other resins [3,19].

This review provides an overview on the state of the art of research on lignin in wood-related applications, as well as on the implementation of lignin-based wood products in the industry.

## 2. Composition of Lignin

The chemical structure of lignin varies significantly depending on the lignin source. Thus, upon considering lignin for any application, the characteristics of the different lignins have to be known, and the right type of lignin has to be carefully selected based on the target application.

Natural lignin is mainly built from three monomers, *ρ*-coumaryl alcohol (H-lignin unit), coniferyl alcohol (G-lignin unit) and sinapyl alcohol (S-lignin unit). The ratio between these monomers differs among lignins of different plant species and in different parts of the plants [7,20]. As such, the lignin in hardwood contains a higher quantity of S-lignin units, while softwood lignin contains more G-lignin units. H-lignin units are less abundant in wood, they can mainly be found in other plants [21,22]. The most common bond connecting the monomers in all natural lignins is the β-*O*-4 ether bond [4,6]. About 45–50% of the bonds in softwoods, and 60–62% in hardwoods are β-*O*-4 ether bonds [7].

For application in wood material treatment G- and H-lignin units and thus softwood or grass lignin is widely considered most promising. The reason for this can be found in the structure of the monomers; G- and H-lignin units have more reactive sites at the benzene ring, making them more reactive toward other binding material or chemical treatment [21,22]. Despite this, recent results have indicated that the lower molecular weight and other characteristics of hardwood lignin molecules might compensate for the lower number of reactive sites, thus producing lignin-phenol-formaldehyde (LPF) resins with similar characteristics. This topic is still under discussion, and a conclusion cannot be given [23,24,25].

Chemical extraction or treatment of lignin leads to even more variance between different lignins. In general, it was found that harsher treatment conditions lead to more significant changes of the structure, up to a point where the structure of these lignins, which are called technical lignins, does not have much in common with the structure of the naturally occurring lignin [7].

One of the most prominent examples of this is the Kraft process for production of chemical pulp. Due to high temperature and alkaline conditions, the structure of the lignin is dramatically changed during the process. The resulting Kraft lignin is a recalcitrant material, which is less reactive than natural lignin. Yet a lot of research on the usage of Kraft lignin has been carried out up to date [7,26]. However, there are also other important processes, during which lignin is extracted as a side product.

In the sulfide process, the lignocellulosic material is treated similarly to the Kraft process, but in an acidic medium. Lignins from the sulfide process are called lignosulfonates. They often have a higher molecular weight than Kraft lignin and are soluble in water due to the sulfonation at the α-position [9]. Due to the high amount of active sulfonic acid groups lignosulfonates are suitable for various potential applications such as dispersants or emulsifiers [27].

Soda pulping is a pulping procedure during which no sulfur is used. It is the main pulping method for non-wood species. The main difference between soda pulping lignin and Kraft lignin is the lower sulfur content [2,9].

Because of the inert and recalcitrant structure of the aforementioned technical lignins, the “lignin-first” approach has received increasing attention. The idea of the approach is to cleave the lignin in a way that valuable products are gained. This could, for example, be achieved via solubilizing lignin with organic solvents [28]. Lignin that is cleaved from the lignocellulosic materials and dissolved by organic solvents is known as organosolv lignin. Usually, organosolv lignin is closer in structure to natural lignin, compared to other technical lignins [9]. However, this largely depends on the severity of the extraction. The solvent also plays a significant role in the yield and composition of the product [29,30].

A more in-depth description on the different types and sources of lignin can be found elsewhere [9,31].

## 3. Applications of Lignin in Wood

Lignin is of interest for various applications in wood products. Most research has been carried out on the application of lignin as an adhesive. Adhesives are used for both solid wood and wood materials, as well as in engineered wood products such as fiberboards. For this application cleaving the lignin is not necessary because adhesives do not have to diffuse into the cell wall [32].

Cleaving the lignin is necessary for a different application, for which lignin-derived substances have high potential. Resins are commonly used for chemical wood modification. Chemical wood modification aims to improve important wood properties like dimensional stability, resistance to moisture, and bio-degradation, etc. To modify the wood, the modification material must enter the cell walls of the wood, and not just the wood lumen [33,34]. The cell walls can be entered from the lumen through micropores, which upon swelling have a maximum diameter of 2–4 nm in a water-saturated state [34]. Because of this diameter only smaller molecules can enter the cell walls [35,36]. As to be expected for a polymer, lignin molecules are too large for this. Because of this, lignin has to be cleaved for applications in chemical wood modification. Additional reasons for cleaving the lignin are the increase of reactive sites, a potential decrease of steric hindrance [37], as well as the potential production of other valuable chemicals, such as vanillin [8].

## 4. Applying Lignin without Cleavage

### 4.1. General Remarks

Considering the branched and stable structure of lignin, the most energy-efficient solution for applying lignin technically would be to apply it without cleavage. As mentioned before, lignin can be used without cleavage for adhesive applications.

Generally, lignin has a lower reactivity than monomers commercially used for resins. The reason for this is steric hindrance as well as additional substitutes on potential reactive sites and on the phenolic hydroxy group. The numbers of reactive sites for different lignins can, for example, be in the range of 0.81–1.72 mmol∙g^−1^, depending on the lignin source [38]. This results in longer pressing times or higher temperatures needed for the curing of the resins containing higher amounts of lignin [39]. Due to this, methods for pretreatment of lignin to improve the reactivity of the lignin and thus the energy efficiency are necessary.

### 4.2. Application in PF Resins

PF resins can be classified into resole-type and novolac-type PF resins. Resoles are formed under alkaline conditions and in excess of formaldehyde, while novolacs are formed under acidic conditions and in excess of phenol. While there are some publications on substituting phenol in novolacs with lignin [40,41,42], to the authors’ knowledge, lignin-containing novolacs have not been applied in wood applications up to date.

#### 4.2.1. Methylolated Lignin in Resoles

To improve the reactivity and thus the feasibility of lignin, various pretreatment methods are used [2,37]. An important method for pretreatment of lignin is methylolation. The aim of methylolation is to add functional hydroxymethyl groups to the lignin so that the lignin can directly react with the polymeric network of the resin. Herein, usually the reaction of formaldehyde with lignin under alkaline conditions is applied [37].

Kalami et al. (2017) managed to produce a resin made from methylolated lignin and formaldehyde, completely replacing the phenol. Timber joints produced with this adhesive exhibited shear lap strengths that were in accordance with the respective national requirements [43]. In a follow-up study, lignins from different sources were compared, showing that lignin containing more H- and G-lignin units are more suitable to replace phenol. The obtained resins performed only slightly worse than the PF resin, even with full replacement of phenol, while decreasing the formaldehyde consumption by up to 50% [22].

The formaldehyde emission of lignin-containing resins has to be examined with care in both PF and UF resins. Several publications reported higher formaldehyde emissions, due to the lower reactivity of the lignin compared to other monomers [44,45,46], while others reported similar or even lower formaldehyde emissions [47,48,49,50] upon replacing part of the phenol or urea monomers with lignin. This indicates that the formaldehyde emission is highly dependent on the ratio of monomers to formaldehyde, and on the reaction conditions.

Ghorbani et al. compared the influence of 20–40% of different types of technical lignins (organosolv, soda, Kraft and lignosulfonates) on PF resins. It was found that out of the given samples pine Kraft lignin and lignosulfonates were the most suitable lignins for replacing phenol. Beech veneer plywood produced with an LPF resin with 20–40% content of pine Kraft lignin even surpassed the tensile shear strength and wet tensile shear strength of PF resin. However, a higher curing temperature was applied to obtain these good properties [21,51]. In a follow-up study, LPF with 40% pine Kraft lignin was applied as an adhesive for beech plywood. The shear strength of the plywood was significantly lower than that of the PF, however, the plywood was suitable for exterior applications according to EN 314-1 [18]. Similarly, another publication reported that at 50% replacement of phenol by Kraft lignin the shear strength and especially the wet shear strength of plywood produced with LPF were inferior to those with PF resin [52]. These results were contradicted by another study, in which up to 50% Kraft lignin led to similar or even slightly improved performance compared to PF-bonded plywood [53]. Even higher substitution levels were reported by Abdelwahab et al. (2011), where up to 90% of phenol was substituted with Kraft lignin. The adhesives were tested according to ASTM D 2339-94A, and the adhesive strength was significantly higher for lignin-containing resins than for control PF resin, even at higher lignin concentrations [54].

In contrast to the results published by Ghorbani et al., Cheng et al. obtained very good results using organosolv lignin in a PF resin. Even at 75% replacement of phenol by organosolv lignin extracted from pine saw dust, the shear strength and the wet shear strength of plywood produced with the LPF resin were higher than those of a reference PF resin [55]. The high potential of organosolv lignin was confirmed in another study, where up to 70% replacement of phenol by the organosolv lignin “Biolignin^TM^” by the company CIMV led to increased shear strengths compared to reference PF resin in plywood [56].

Eucalyptus plywood was produced with PF resin in which phenol was substituted with 5–25% enzymatic hydrolysis lignin from cornstalk residues from bio-ethanol production extracted with sodium hydroxide solution. The plywood almost met the requirements for first grade plywood according to the Chinese National Standard (GB/T 14732-2006). The best results were achieved with 10% phenol substitution [57]. Another study using enzymatic hydrolysis lignin indicated that in adhesives the substitution of up to 50% of the phenol is possible without inferior mechanical performance. A shear strength of 1.05 MPa was reported for three-layered plywood at 10% substitution of phenol with lignin [58]. A similar shear strength was reported for plywood made with a resin of 25% phenol, 25% urea and 50% unpurified bio-ethanol fermentation residues. Three-layer plywood bonded with this resin met the Chinese national standards according to GB/T 17657-1999 [59].

Another comparison between LPF made with different lignins indicated that alkaline-extracted lignin was most suitable out of alkaline-extracted, Kraft, organosolv and enzymatic hydrolysis lignin, at a substitution rate for 20% phenol. Particle boards produced with Kraft lignin containing LPF were suitable for P6 applications according to DIN EN 312:2010-12; the particle boards produced with enzymatic hydrolysis lignin, alkaline-extracted lignin and organosolv lignin containing LPFs performed well enough for P5 applications [25]. In another study on particle boards with PF resins, it was reported that 20–30% of the phenol could be substituted with organosolv lignin without a significant effect on the properties of the particle board [60].

In a comparison between lignin separated from different biomasses, including sugar mill bagasse, coconut coir, eucalyptus bark, and coffee beans using sodium hydroxide on small scale, each of the tested lignins improved the mechanical performance of wood samples bonded with PF resin up to 50% substitution tested according to IS 851:1978 [61].

#### 4.2.2. Other Pretreatment Methods for Lignin in Resoles

Phenolation is the condensation of phenol and lignin, providing more reactive sites as well as cleavage of the ether bonds [37,38]. Plywood panels made with LPF with phenol substituted with 33% phenolated lignin and formaldehyde performed worse than plywood made with PF resin or plywood made with LPF resin with methylolated lignin [62].

The preparation of methylolated nanosized alkalilignin could be interesting for application in PF resins, since it can be done in mild conditions by acidic precipitation from ethylene glycol. Tests of the performance of plywood produced with nanolignin-PF resins indicated a higher bond strength and wet bond strength at similar or slightly lower formaldehyde emission compared to normal PF resins. The highest bond strengths and wet bond strengths were achieved at 30 and 40% substitution of phenol with nanolignin [48]. In another study 5 or 10% of nanosized and microsized alkali lignin was added to a PF resin, with the best properties being achieved with 5% nanolignin. The shear strength of wood lap joints with the resin could be increased from 8.7 MPa (pure PF resin) to 10.9 MPa [63].

The modification of the pine wood Kraft lignin by Fenton-oxidation and Fenton-oxidation with subsequent ammoxidation did not improve the performance of LPF resins as wood adhesives [64].

Substituting 30% of phenol with demethylated soda lignin in an LPF resin considerably decreased the performance of plywood produced with the resin. Only lignin demethylated with Na_2_SO_3_ could be promising for future applications [65].

#### 4.2.3. Comparison of Selected Examples

For better comparison, selected examples of different PF resins, the wood sample and the mechanical properties measured for it are presented in Table 1.

### 4.3. Application in UF Resins

Because of the similarity in structure, the application of lignin in PF resins is the first that comes to mind; however, lignin can also form bonds with other resins. The most prominent example of this is the formation of lignin-urea-formaldehyde (LUF) resins, because UF is the most produced adhesive for wood applications worldwide [66].

Addition of up to 20% of phenolated Kraft lignin only slightly decreased the internal bond strength of a UF resin in a particle board while significantly lowering the formaldehyde emission of the board [47].

Lignin can be pretreated with ionic liquids, which separates the lignin from lignocellulosic biomass. Treatment with ionic liquids decreases the quantity of methoxy-groups, thus increasing the reactivity of lignin [1]. Replacing urea in UF resins by soda bagasse black liquor treated with ionic liquids led to a decrease in shear strength and wood failure in plywood. The decrease in shear strength and wood failure was less pronounced than for samples with the same amount of untreated black liquor, confirming the increase of reactivity [67]. In a follow-up study, the performance of the LUF resins could be improved by the addition of 6% of the resin weight of polymeric diphenylmethane diisocyanate (PDMI). However, a direct comparison to UF resin was not described [68].

Another pretreatment method for the application of lignin in UF resins was recently presented by Gao et al. (2020) [49] for the application as an additive in plywood and medium-density fiberboards (MDFs). Methylolated lignin was esterified with maleic anhydride. The addition of 5% of maleic anhydride-treated lignin to a UF resin for plywood applications performed comparatively as well as plywood treated with reference UF resin while additionally decreasing the water absorption of the plywood [49].

The storage time of UF resin could be increased from 30 d to 200 d with addition of 20% methylolated sodium lignosulfonate. The formaldehyde emission and shear strength of eucalyptus plywood cured with the modified resin only slightly changed compared to pure UF resin. However, at 60% substitution, the properties were significantly worse [46].

For better comparison, selected examples of different UF resins, the wood sample and the mechanical properties measured for it are presented in Table 2.

### 4.4. Application in Other Resins

Glyoxal was used to synthesize a formaldehyde-free lignin-urea-glyoxal (LUG) resin. The mechanical properties and the water resistance of the LUG resin were worse than those of resins with formaldehyde; however, they could be improved by adding nanoclay or epoxy resin. The best results were achieved with 5% of the weight of the resin addition of epoxy resin. The wood failure in dry shear strength tests was 100% at 1.7 MPa, the wet shear strength was 1.3 MPa with 80% wood failure [69,70]. Additionally, research on the use of glyoxal in lignin-phenol-glyoxal (LPG) resin with black liquor from soda bagasse in particle boards was done. The particle boards performance was inferior to a reference PF resin; however, the requirements for the related EN-standards were still fulfilled [71]. In a follow-up study, the performance of the resin was improved by adding 7% of the resins’ weight of epoxy resin. Plywood bonded with this resin had shear strength, wet shear strength and water absorption comparable to plywood produced with reference PF resin [72].

Another approach was proposed by Zhang et al. (2019) [10], who described a lignin-furfuryl-glyoxal adhesive. Adding up to 9% of the resins’ weight of epoxy resin to improve the water resistance, the adhesive was used for beech wood particle boards. While the internal bond (IB) strength, the wet IB strength and the curing time of the particle boards were worse than with a commercial PF resin, they still met the Chinese regulation requirements according to GB/T17657(2006) [10].

A method to increase the water resistance of LPF resins is to add furfural. Resins made with 50% phenol replacement by Kraft lignin and 15% addition of furfural were used to produce plywood. The mechanical properties were similar to plywood produced with reference PF resin [73].

Another field is the use of lignin in polyurethane adhesives. Lignin can improve the mechanical performance of polyurethanes when used as a polyol. Enhancing this effect even further can be achieved by using demethylated lignin. A total of 20% of polyethylenglycol in a polyurethane system with toluol-diisocyanate was replaced by the lignin. A tensile strength of 91.2 MPa was reported for the adhesives binding single aspen wood chips according to GB/T 9846-2015 [74]. A self-healing, recoverable polyurethane adhesive with 20% lignin modified with long chain polyetheramines was presented by Liu et al. (2020) [75]. The polyurethane adhesives all showed improved lap shear strengths upon addition of lignin or modified lignin, the best value being a lap shear strength of 7.8 MPa for beech wood boards in the first test (upon reusing, even higher values were achieved) [75].

Ammonium lignosulfonate treated with H_2_O_2_ and blended with polyethyleneimine was used as a binder in the production of MDF. Under optimal conditions (170 °C hot pressing temperature, 7 minutes pressing time, 20% binder content), the Chinese requirements for mechanical properties of MDF were met [76].

Other pretreatment methods and observations that were described in literature will be mentioned briefly below. Oxidation of lignin by laccase increases the reactivity [77]. Solvent fractioning can be used to obtain higher and lower molecular weight fractions from Kraft lignin. The results indicated that the mechanical properties of a cured LPF resin are not significantly influenced by the molecular weight of the lignin [15]. The reactivity of fractionated Kraft lignin can be improved by phenolation. However, plywood produced with an LPF resin with 40% phenolated Kraft lignin fractionated by solubilization in different solvents was inferior to plywood produced with PF resin for all fractions. Despite this, the properties of the plywood met the Chinese national standards GB/T14732 [44]. Lignin can function as a fire retardant and photo stabilizer in epoxy resins [78,79].

Selected examples for different resins as well as their mechanical properties are listed in Table 3.

## 5. Cleavage of Lignin

### 5.1. General Remarks

Most publications on lignin in wood applications deal with uncleaved lignin. However, for wood modification, the cleavage of lignin is inevitable. Hence, the cleavage of lignin and applications of products thereof will be described.

### 5.2. Characterization of Cleavage Products

There are various ways to cleave lignin, e.g., hydrolysis, pyrolysis, liquefaction, etc. However, in most lignin depolymerization methods, the main products are phenolic compounds such as phenol, catechol, guaiacol, ortho- and para-cresol, etc. [80,81]. A screening of the combability of these monomers with PF-resols was done by Biziks et al. (2020) [80]. Herein, it was reported that guaiacol, o-, m-, p-cresol, and 4-ethylphenol were suitable for phenol replacement, whereas catechol, 4-methylguaiacol, and syringol had negative impacts on the performance of the resin. Bio-oils obtained by cleaving lignin contain many compounds that are suitable for substitution of phenol in PF resins. However, due to the complex mixture it is often not possible to obtain a bio-oil that does not also contain molecules that worsen the resins’ characteristics [80].

Dier et al. (2017) [82] tested the influence of model bio-oils with different ratios of monomers on the characteristics of the resols. As in the study by Biziks et al. (2020), it was found that bio-oils that contain cresols have high potential to replace phenol in PF resins, while catechols negatively impacted the properties of the resin [82].

### 5.3. Cleavage of Lignin

In literature, pyrolysis is the most described method for biomass cleavage. However, despite the great amount of research, the application of pyrolysis oil of lignin for wood applications has received little attention. A reason for this could be that, considering the current price for lignin, the on-site production of bio-oils from technical lignin is in most cases not economically feasible, as was shown in a study by Farag et al. [83]. An LPF resin with 0–100% phenol substitution with wheat straw lignin depolymerized by alkali-catalyzed microwave digestion was prepared. The bonding strength of a plywood was higher with 40% substitution than for reference PF resin, and it was similar even at 80% substitution [84].

Organosolv lignin from pine sawdust cleaved in hot water/ethanol under high pressure and hydrogen atmosphere with metallic salt-catalyst-produced monomers, which were used to replace up to 75% of phenol in a PF resin. At both 50 and 75% replacement, the wet and dry shear strength of plywood bonded with the resin could be improved, while only at 75% replacement the moisture resistance decreased. The mechanical properties were, however, not better than those reported for a PF resin with non-degraded organosolv lignin prepared by the same method [55].

Amounts of 50–70% of phenol in a PF resin were replaced by base-catalyzed depolymerized pine Kraft lignin. The longitudinal tensile shear strength of wood joints produced with the resin was similar to or higher than that of the reference PF resin. The best results were achieved with 70% replacement of phenol [85].

Resins made from different lignins liquefied with phenol and inorganic acid catalysts were tested for application in PF resins. The highest dry and wet shear strengths were achieved with alkaline lignin, HCl as a catalyst, and with a phenol replacement of 40%. The mechanical properties were similar to those of commercial PF resin, with an increased F emission. Lauan plywood produced this way had a tensile shear strength of 1.42 MPa and a warm-water-soaked binding strength of 1.22 MPa [45].

Selected examples of resins containing cleaved lignin products and their mechanical properties are listed in Table 4.

### 5.4. Cleavage of Lignocellulosic Biomass

Upon producing bio-oil from natural lignin, the lignin is often not separated from the lignocellulosic material, as this would be an additional energy-consuming work step. Instead, the biomass is often directly treated. However, because this review focuses mainly on separated lignin, the cleavage and usage of lignocellulosic biomass will only be briefly discussed. In literature, the water-insoluble part of bio-oils derived from biomass are often referred to as “pyrolytic lignin”, as they mainly contain lignin-derived compounds and thus resemble lignin pyrolysis oil well [86].

In a patent formulated in 1999, pyrolysis oil of wood bark, a side product in forestry, was substituted for 40% of the phenol in PF resins for oriented strand boards. The internal bonding and the torsion shear strength increased compared to a commercial PF resin [87]. Similarly, 30% of phenol was replaced by pyrolysis oil of wood bark in another study. The properties of oriented strand board (OSB) with this resole, produced with standard procedures commonly used in industry, met the Canadian standards CSA (0437.1–93) [88]. In another study, 40–50% of phenol in a PF was replaced by pyrolysis oil from pure wood or bark for plywood and OSB production. Similar or better performance compared to plywood and OSB with control PF resin was achieved under industrially common production conditions and at larger scale production [81].

Pyrolysis of wood residues has the problem of there being additional pyrolysis products from cellulose and hemicelluloses that do not react in the resin and thus might weaken it [89,90]. Hence, distillation and purification of the fast pyrolysis products of wood wastes with the organic solvents hexane or benzene can significantly improve the water resistivity of the resin. For an LPF resin with 40% substitution of phenol, both a dry and a wet shear strength of about 3.25 MPa were measured in wood glue line tests [89]. Separating the fast pyrolysis products by water and methanol extraction produced a pyrolytic lignin that could replace up to 40% of the phenol in PF resin for the application in OSB. At 30% substitution, the bio-oil containing OSB had comparable modulus of rupture (MOR), modulus of elasticity (MOE), TS, water absorption and dry or wet internal bond strengths to the reference OSB with PF resin [90].

Celikbag et al. [91] replaced different amounts of phenol in a PF resin with bio-oil from hydrothermal liquefaction of sweetgum wood chips without bark. The bonding strength of the obtained resin tested on thin wood stripes increased from 6.3 MPa (PF resin) to 7.8 MPa for PF resin with about 75% of phenol replaced by the bio-oil [91].

Applying the cleaved products of biomass as a whole in wood applications has received far more attention than those of lignin specifically. This shows a potential lack of research on the application of lignin cleavage products in wood applications. Future research in this field could lead to important new insights and discoveries.

## 6. Challenges for the Application of Lignin and Lignin-Derived Compounds

The high amount of research indicates the interest in the valorization of lignin; however, commercial applications have been limited up to date. Reasons for this are different challenges that have to be met. The low reactivity of lignin is a challenge that can be overcome by lignin modification or cleavage, but also by higher curing temperatures, pressing time or accelerators [37,39]. However, further chemical modification increases the cost of lignin utilization. This is especially a problem because several reports have indicated that currently the utilization of lignin is not economically feasible [83,92,93]. Another challenge is the heterogeneity of lignin, due to which lignin often shows unexpected behavior [8,27]. Technical lignins obtained from different plant species have different properties, and different ratios, for example, in black liquor, can lead to varying quality in the final application [22,23]. An answer to that challenge might be lignin fractionation or cleavage [15,44]. In the field of wood modification the size of the lignin molecules is problematic, as only small molecules can enter the cell wall [34,35,36]. Methods for lignin cleavage to use lignin for such applications are underway. However, not all lignin cleavage products are suitable for the substitution of monomers commonly used in wood modification [80,81].

## 7. Current or Past Commercial Products of Lignin-Based Adhesives in Wood Industry

As mentioned before, industrial commercial products of lignin have been limited up to date. Until now, lignin has only been sold as an adhesive; products for wood modification are, to the authors’ knowledge, not commercialized. Nevertheless, there are some examples of companies who have offered or are offering lignin-based wood products.

In the past, particle boards and fiberboards with an LPF resin containing 25% lignin were produced by the company VEB Plasta Erkner (today Prefere Resins Holding GmbH). The production of these products was stopped in 1990 because the black liquor used as the lignin source could not be provided anymore, and substituent lignin sources performed worse [94,95]. However, the company did not lose interest in lignin, and in 2017 the production of LPF resin with 50% lignin was started, and fiberboards as well as other wood-based materials treated with the resin are on the market [95]. Interestingly, in the same year also the company UPM Biochemicals commercialized an LPF product. UPM BioPiva^TM^ allows to replace 80% of phenol for the production of plywood and other wood products [96]. Stora Enso is another company that offers lignin products. Their Lineo^TM^ Kraft lignin is, as they advertise, suitable for replacing phenols in resins for plywood, oriented strand board and laminated veneer lumber [12]. Lignins as a replacement for resins in fiberboards are also offered by Lignotech, a company owned by Borregaard [97]. The French company CIMV provides the organosolv lignin “Biolignin^TM^”, which performs well as an adhesive for plywood with replacement of up to 70% of phenol in PF resin [13,56]. There are several companies that are offering or currently implementing further lignin-based products for wood treatment [14,98,99,100].

These examples show that the commercial application of lignin as an adhesive for wood is on the rise. This indicates that the research on lignin is, at least in this area, close to the needs of industry. This was confirmed by an importance performance analysis, wherein the majority of experts from industry and research stated that the research on lignin application in PF resins is promising in the most relevant aspects [101]. The rising commercial interest as well as the rising lignin price and lignin market further underline this [93,102].

Additional indications of high commercial interest in lignin applications are patents. There are several patents describing resins containing lignin [87,103,104,105,106,107,108,109]. However, most of the patents mentioned herein were assigned a long time ago, so implications for the recent interest in lignin cannot be derived from them.

Until now, there are only a few life cycle assessment studies on lignin-derived wood products [3]. This can be a problem, as in some cases the new products have a similar or even slightly worse sustainability compared to classical solutions [110]. However, in the majority of the studies the results have indicated that the substitution of resin monomers with lignin improves the sustainability of wood products [110,111,112].

## 8. Conclusions

Up to date, there have only been few examples of the commercial application of lignin as an adhesive and none for the application of lignin for wood modification. In contrast, in the scientific field there are many reports on lignin application as an adhesive, and several reports on the use of lignin in wood modification. While many of the herein proposed lignin-based products fail to achieve the same mechanical properties as commercial resins or are not commercially feasible, there is still a discrepancy between the theoretical knowledge and the practical applications. A large variety of promising methods and approaches have been presented, and the number of recent publications shows that the scientific interest and ability to introduce new ideas is still high. Additionally, several studies have suggested that the substitution of commercial monomers with lignin would have environmental benefits [110,111,112]. However, in some publications it has been stated that currently, due to the low phenol prices, the use of lignin is not economically feasible [92,93]. The bottleneck could be the transition from laboratory to industrial scale. To improve this, more communication and projects combining science and industry are necessary. The rising number of companies that are developing new lignin-based methods or products indicates that this process is already happening. Hence, there is a likelihood that lignin will in the future fulfill its potential to a higher degree.

## Figures and Tables

**Table 1:** Resin composition, sample type, and mechanical properties of selected PF resins described in literature.

Phenol Substitution Levels in the Resin	Wood Sample	Mechanical Properties	Reference
20% substitution with methylolated organosolv lignin	Particle board	Internal bond strength (IB) = 1.02 MPa, Thickness swelling (TS) (2 h) = 10.6%	[60]
PF resin with 5% nanolignin	Wood lap joints	Shear strength = 10.9 MPa	[63]
100% substitution with methylolated enzymatic hydrolysis lignin	Plywood	Dry shear strength: 3.4 MPa (84% wood failure), wet shear strength: 2.6 MPa (73% wood failure)	[43]
40% phenolated sodium lignosulfonates	Plywood	Shear strength = 5.6 MPa	[51]
Lignin-Formaldehyde resins with methylolated softwood Kraft lignin, and corn stover enzymatic hydrolysislignin	Plywood	Enzymatic hydrolysis lignin: shear strength = 3.2 MPa (75% wood failure), softwood Kraft lignin: shear strength = 2.6 MPa (41% wood failure)	[22]
50% replacement with methylolated pine sawdust organosolv lignin	Plywood	Tensile strength = 2.25 MPa (93% wood failure), tensile wet strength = 1.9 MPa (85% wood failure)	[55]
PF resin with 30% nanolignin	Plywood	Dry bond strength = 1.59 MPa (100% wood failure), wet bond strength = 0.89 MPa (100% wood failure)	[48]

**Table 2:** Resin composition, sample type, and mechanical properties of selected UF resins described in literature.

Urea Substitution Levels in the Resin	Wood Sample	Mechanical Properties	Reference
10% ionic liquid pretreated soda bagasse lignin	Plywood	shear strength = 1.89 MPa, wood failure = 70%	[67]
5% lignin-based polyacid catalyst	Plywood and MDF	Plywood: shear strength = 1.72 MPa, wet shear strength = 1.2 MPa MDF: IB = 1.35 MPa, 24h TS = 9%	[49]
15% ionic-liquid-treated soda bagasse lignin with 6% PMDI additive	Plywood	Dry shear strength = 2.2 MPa (100% wood failure), wet shear strength = 0.99 MPa (70% wood failure), water adsorption = 22%	[68]

**Table 3:** Resin composition, sample type, and mechanical properties of selected resins on different basis than PF and UF described in literature.

Resin Composition	Wood Sample	Mechanical Properties	Reference
LPG resin with 20% soda bagasse black liquor	Particle board	IB = 0.6 MPa, water absorption = 14%, thickness swelling = 10%	[71]
LPG resin with 30% bagasse soda black liquor and 7% epoxy resin	Plywood	Dry shear strength = 1.74 MPa (100% wood failure); wet shear strength = 1.0 MPa (80% wood failure), thickness swelling = 4%	[72]
LUG resin with 15% glyoxalated bagasse soda black liquor and 5% epoxy resin	Plywood	Dry shear strength = 1.7 MPa (100% wood failure), Wet shear strength = 1.3 MPa (80% wood failure), Water absorption = 6%	[70]
Polyurea adhesive doped with 20% polyetheramine chain grafted lignin with functional disulfide bonds	Beech wood boards	Lap shear strength = 7.8 MPa	[75]

**Table 4:** Resin composition, sample type, and mechanical properties of selected resins with cleaved lignin described in literature.

Resin Composition	Wood Sample	Mechanical Properties	Reference
PF resin with 70% oligomer lignin from pine Kraft lignin treated by base-catalyzed depolymerization	Beech lamellas	Tensile shear strength = 15 MPa. Wet tensile shear strength about 6 MPa	[85]
PF resin with 40% alkaline-catalyzed microwave-digested wheat straw lignin	Plywood	Bonding strength = 1.70 MPa	[84]
PF resin with 40% phenol replacement by lignin liquefied in phenol	Plywood	Dry bonding strength = 1.42 MPa, warm water soaking bonding strength = 1.22 MPa, repetitive boiling water soaking bonding strength = 1.10 MPa	[45]
